# Supplementary material for: Bootstrap simulations for evaluating the model estimation of the extent of cross-pollination in maize at the field-scale level
Source: PLoS One. 2021 May 19;16(5):e0249700. doi: 10.1371/journal.pone.0249700 (PMC8133429; doi:10.1371/journal.pone.0249700)
Supplement: S1 Table — (DOCX) [file pone.0249700.s001.docx]

**S1 Table. Regression parameters, deviance, and AIC for the complete dataset in models with parameter *P*_0_.**

| Model | *P*_0_ | a | b | deviance | AIC |
| --- | --- | --- | --- | --- | --- |
| CP_4_ | 0.6423 | 0.0835 | -0.3886 | 50325 | 68626 |
| CP_5_^a^ | 0.2745 | 0.1 | 0.2 | 73118 | 91415 |
| CP_6_^a^ | 0.2440 | 0.1 | 0.2 | 83639 | 97083 |
| CP_7_ | 0.7611 | 0.1330 | -0.4586 | 51038 | 64485 |

*a*: parameter estimation for CP_5_ and CP_6_ suggested by reference [12].
