# Supplementary material for: Bootstrap simulations for evaluating the model estimation of the extent of cross-pollination in maize at the field-scale level
Source: PLoS One. 2021 May 19;16(5):e0249700. doi: 10.1371/journal.pone.0249700 (PMC8133429; doi:10.1371/journal.pone.0249700)
Supplement: S2 Table — (DOCX) [file pone.0249700.s002.docx]

**S2 Table. Deviance and AIC for models with parameter *P*_0_ in the calibration and validation sets.**

| Model | Cross-Pollination Model^a^ | Calibration Set |  | Validation Set |  |
| --- | --- | --- | --- | --- | --- |
|  |  | **Deviance** | **AIC** | **Deviance** | **AIC** |
| CP_4_ | $CP=0.7017\times{10}^{\left[ 0.0769\sqrt{FB}+(-0.3963)\sqrt{distance} \right]}$ | 33588 | 45629 | 17442 | 23719 |
| CP_5_ | $CP=0.2747\times{10}^{\left[ (-0.1)\sqrt{FB}+(-0.2)\sqrt{distance} \right]}$ | 49798 | 61835 | 25257 | 31530 |
| CP_6_ | $CP=0.003+0.2438\times{10}^{\left[ (-0.1)\sqrt{FB}+(-0.2)\sqrt{distance} \right]}$ | 57062 | 65785 | 27456 | 33728 |
| CP_7_ | $CP=0.003+0.8382\times{10}^{\left[ 0.1278\sqrt{FB}+\left( -0.4692 \right)\sqrt{distance} \right]}$ | 33860 | 42587 | 16180 | 22456 |

*a*: cross-pollination (CP) rate (%).
