# Supplementary material for: Bootstrap simulations for evaluating the model estimation of the extent of cross-pollination in maize at the field-scale level
Source: PLoS One. 2021 May 19;16(5):e0249700. doi: 10.1371/journal.pone.0249700 (PMC8133429; doi:10.1371/journal.pone.0249700)
Supplement: S3 Table — (DOCX) [file pone.0249700.s003.docx]

**S3 Table. Deviance, AIC and r for models with parameter *P*_0_ based on the simulation data.**

|  | Calibration Set |  | Validation Set |  |  |
| --- | --- | --- | --- | --- | --- |
| Model | **Deviance** | **AIC** | **Deviance** | **AIC** | **r** |
| CP_4_ | 33474 ± 1281 | 45679 ± 1329 | 16794 ± 903 | 22897 ± 937 | 0.759 ± 0.024 |
| CP_5_ | 48743 ± 2774 | 60944 ± 2813 | 24314 ± 1845 | 30413 ± 1873 | 0.645 ± 0.023 |
| CP_6_ | 55758 ± 3018 | 64720 ± 3060 | 26551 ± 1951 | 32650 ± 1977 | 0.645 ± 0.023 |
| CP_7_ | 33944 ± 1236 | 42910 ± 1296 | 15666 ± 835 | 21768 ± 866 | 0.776 ± 0.026 |

***a***: mean ± standard deviation.
