# Supplementary material for: Bootstrap simulations for evaluating the model estimation of the extent of cross-pollination in maize at the field-scale level
Source: PLoS One. 2021 May 19;16(5):e0249700. doi: 10.1371/journal.pone.0249700 (PMC8133429; doi:10.1371/journal.pone.0249700)
Supplement: S1 File — (DOCX) [file pone.0249700.s004.docx]

S1 File

The original CP rate (%) of Table 1 in various rows and the distances from the pollen source in the 2009-1, 2009-2, and 2010-1 fields are available from

<https://figshare.com/articles/dataset/CP_data_xlsx/13370756>

where

distance= distance from the pollen source (m)

CP= cross-pollination rate

Po= the CP rate (%) at the edge of the pollen recipient field

ID= isolation distance
