# Supplementary material for: Bootstrap simulations for evaluating the model estimation of the extent of cross-pollination in maize at the field-scale level
Source: PLoS One. 2021 May 19;16(5):e0249700. doi: 10.1371/journal.pone.0249700 (PMC8133429; doi:10.1371/journal.pone.0249700)
Supplement: Attachment — Submitted filename: Response_to_reviewer(20201004).docx [file pone.0249700.s006.docx]

1. Please ensure that your manuscript meets PLOS ONE's style requirements, including those for file naming. The PLOS ONE style templates can be found at

https://journals.plos.org/plosone/s/file?id=wjVg/PLOSOne_formatting_sample_main_body.pdf and

https://journals.plos.org/plosone/s/file?id=ba62/PLOSOne_formatting_sample_title_authors_affiliations.pdf

**Response:**

Thanks for the reminder from the editorial office. The manuscript has been checked follow the PLOS ONE's style requirements.

2. Please upload a new copy of Figure 5 as the detail is not clear. Please follow the link for more information: https://blogs.plos.org/plos/2019/06/looking-good-tips-for-creating-your-plos-figures-graphics/" https://blogs.plos.org/plos/2019/06/looking-good-tips-for-creating-your-plos-figures-graphics/

**Response:**

Thanks for the reminder from the editorial office. The new copy of figure 5 with higher resolution has been upload.

3. We note that you have included the phrase “data not shown” in your manuscript. Unfortunately, this does not meet our data sharing requirements. PLOS does not permit references to inaccessible data. We require that authors provide all relevant data within the paper, Supporting Information files, or in an acceptable, public repository. Please add a citation to support this phrase or upload the data that corresponds with these findings to a stable repository (such as Figshare or Dryad) and provide and URLs, DOIs, or accession numbers that may be used to access these data. Or, if the data are not a core part of the research being presented in your study, we ask that you remove the phrase that refers to these data.

**Response:**

Thanks for the reminder from the editorial office. In our manuscript, the data referred by the phrase “data not shown” in LINE 392 and LINE420 are not a core part of the research being presented in our study, and in LINE 443, the value of r could be calculated by Table 3. Therefore, the phrase “data not shown” has been removed.

4.Thank you for stating the following in the Acknowledgments Section of your manuscript:

[This research is partially supported by the Ministry of Science and Technology under Grant Number 108-2634-F-005-003 through “Pervasive AI Research (PAIR) Labs, Taiwan”, and “Innovation and Development Center of Sustainable Agriculture” from The Featured Areas Research Center Program within the framework of the Higher Education Sprout Project by the Ministry of Education (MOE) in Taiwan.].

We note that you have provided funding information that is not currently declared in your Funding Statement. However, funding information should not appear in the Acknowledgments section or other areas of your manuscript. We will only publish funding information present in the Funding Statement section of the online submission form.

Please remove any funding-related text from the manuscript and let us know how you would like to update your Funding Statement. Currently, your Funding Statement reads as follows:

The authors received no specific funding for this work.

Please include your amended statements within your cover letter; we will change the online submission form on your behalf.

We note that one or more of the authors is affiliated with the funding organization (“Pervasive AI Research (PAIR) Labs, Taiwan), indicating the funder may have had some role in the design, data collection, analysis or preparation of your manuscript for publication; in other words, the funder played an indirect role through the participation of the co-authors.

If the funding organization did not play a role in the study design, data collection and analysis, decision to publish, or preparation of the manuscript and only provided financial support in the form of authors' salaries and/or research materials, please review your statements relating to the author contributions, and ensure you have specifically and accurately indicated the role(s) that these authors had in your study in the Author Contributions section of the online submission form. Please make any necessary amendments directly within this section of the online submission form.  Please also update your Funding Statement to include the following statement: “The funder provided support in the form of salaries for authors [insert relevant initials], but did not have any additional role in the study design, data collection and analysis, decision to publish, or preparation of the manuscript. The specific roles of these authors are articulated in the ‘author contributions’ section.”

If the funding organization did have an additional role, please state and explain that role within your Funding Statement.

Please also provide an updated Competing Interests Statement declaring this commercial affiliation along with any other relevant declarations relating to employment, consultancy, patents, products in development, or marketed products, etc.

Within your Competing Interests Statement, please confirm that this commercial affiliation does not alter your adherence to all PLOS ONE policies on sharing data and materials by including the following statement: "This does not alter our adherence to PLOS ONE policies on sharing data and materials.” (as detailed online in our guide for authors [http://journals.plos.org/plosone/s/competing-interests](about:blank)). If this adherence statement is not accurate and there are restrictions on sharing of data and/or materials, please state these. Please note that we cannot proceed with consideration of your article until this information has been declared.

Please know it is PLOS ONE policy for corresponding authors to declare, on behalf of all authors, all potential competing interests for the purposes of transparency. PLOS defines a competing interest as anything that interferes with, or could reasonably be perceived as interfering with, the full and objective presentation, peer review, editorial decision-making, or publication of research or non-research articles submitted to one of the journals. Competing interests can be financial or non-financial, professional, or personal. Competing interests can arise in relationship to an organization or another person. Please follow this link to our website for more details on competing interests: <http://journals.plos.org/plosone/s/competing-interests>

**Response:**

Thanks for the reminder from the editorial office. In cover letter, we would like to update our Funding Statement, because we will only get the financial support for the Article Processing Charge (APC) for our article published. In addition, the funding organization did not have an additional role in the study design, data collection and analysis, decision to publish, or preparation of the manuscript and did not provide financial support in the form of authors' salaries and/or research materials. The academic affiliation does not alter our adherence to PLOS ONE policies on sharing data and materials.

[Note: HTML markup is below. Please do not edit.]

Reviewers' comments:

Reviewer's Responses to Questions

**Comments to the Author**

1. Is the manuscript technically sound, and do the data support the conclusions?

The manuscript must describe a technically sound piece of scientific research with data that supports the conclusions. Experiments must have been conducted rigorously, with appropriate controls, replication, and sample sizes. The conclusions must be drawn appropriately based on the data presented.

Reviewer #1: Yes

Reviewer #2: Yes

2. Has the statistical analysis been performed appropriately and rigorously?

Reviewer #1: I Don't Know

Reviewer #2: Yes

3. Have the authors made all data underlying the findings in their manuscript fully available?

The [PLOS Data policy](http://www.plosone.org/static/policies.action#sharing) requires authors to make all data underlying the findings described in their manuscript fully available without restriction, with rare exception (please refer to the Data Availability Statement in the manuscript PDF file). The data should be provided as part of the manuscript or its supporting information, or deposited to a public repository. For example, in addition to summary statistics, the data points behind means, medians and variance measures should be available. If there are restrictions on publicly sharing data—e.g. participant privacy or use of data from a third party—those must be specified.

Reviewer #1: No

Reviewer #2: Yes

4. Is the manuscript presented in an intelligible fashion and written in standard English?

PLOS ONE does not copyedit accepted manuscripts, so the language in submitted articles must be clear, correct, and unambiguous. Any typographical or grammatical errors should be corrected at revision, so please note any specific errors here.

Reviewer #1: Yes

Reviewer #2: Yes

5. Review Comments to the Author

Please use the space provided to explain your answers to the questions above. You may also include additional comments for the author, including concerns about dual publication, research ethics, or publication ethics. (Please upload your review as an attachment if it exceeds 20,000 characters)

Reviewer #1: This paper studies the dispersal of maize pollen from a pollen source as a function of distance. Three fields are used for study. Seven nonlinear regression models are fitted. A bootstrap analysis is performed to provide confidence limits. Furthermore, cross validation is conducted.

The statistical methodology needs to be more accurately described. Some detailed comments follow.

(1) The study obviously involves a large number of plots per field. But it remains unclear what the dimension of the plots are and how they are distributed across the field. A map showing the field layout would be very useful.

**Response to Reviewers:**

Thanks for the reviewer’s comment. We have added the example of the layout and the dimension of sampling plots in Fig.1.

(2) It is not clear from M&M whether the regression models were fitted per field or across fields.

**Response to Reviewers:**

Thanks for the reviewer’s comment. The PMFG model were fitted across fields. As shown in Table 2, the results of regression parameters, RMSE, and R^2^ were fitted the complete dataset. The statement was also corrected and inserted into LINE 426.

(3) Missing plots were estimated using some clustering method, but it is unclear just why missing values had to be replaced (lines 294-297). Only a very small number or plots was missing, and then nonlinear models look quite simple, so I do no see any need to replace missing values.

**Response to Reviewers:**

Thanks for the reviewer’s comment. In our study, the P_0_, the CP rate (%) of the first row of the pollen recipient field, is required using the PMFG models. However, the collected data will be lost resulted from the environmental conditions of the site. Therefore, to ensure that the PMFG models can be evaluated, the k-nearest neighbor algorithm (k-NN) proposed by Šuštar-Vozlič et al. 2010 [13] does need to be used in our study.

(4) I read lines 260-263 several times, but could not understand the meaning. Can this be reworded? How can the gene flow be reduced by fitting a model? I do not understand.

**Response to Reviewers:**

Thanks for the reviewer’s comment. The statement means that as reported in Gustafson et al. 2006 [12], the trend of gene flow within recipient or border rows could be reduced as represented as the coefficient of the proportionality constant, and twice as effective as the ID (the unplanted isolation buffer). Then, in our study, the proportionality constants for FB and the distance within the recipient field were re-estimated based on the data from field experiments conducted. The statement was also corrected and inserted into LINES 260 to 263.

(5) For the bootstrapping, it is unclear if this was done by field or for all data across lumped together across the three fields.

**Response to Reviewers:**

Thanks for the reviewer’s comment. For the bootstrapping, random sampling with replacement was applied to the actual data set across lumped together across the three fields. The statement was also corrected and inserted into LINE 309.

(6) How was the nonlinear regression done, what procedure in SAS was used? Was regression done by field or across fields? In the latter case, how was heterogeneity between fields taken into account?
**Response to Reviewers:**

Thanks for the reviewer’s comment. In our study, PROC NLIN was used for constructing the PMFG model across the three fields. On the other hand, due to the facts that the three fields were set in the same location, Puzih City, Chiayi County and the trend of gene flow were all similar, three data sets collected from the same field were combined and used to evaluate the performance of the PMFG model.

(7) How was the cross validation done? Which SAS procedure? How was the data split into calibration and validation sets? It would be most meaning full, e.g., to use two fields for calibration and one for validation.
**Response to Reviewers:**

Thanks for the reviewer’s comment. Three data sets collected from the same field were combined as the complete data. Then, the complete experimental data were randomly partitioned into two sets, a calibration set (two thirds of the samples) and a validation set (one third of the samples), for fitting to the model and validating their predictive abilities, respectively.

(8) Strictly speaking the number of true replications is given by the number of fields, whereas the many plots per field constitute pseudo-replications. This limitation of the study deserves to be mentioned and critically discussed.

**Response to Reviewers:**

Thanks for the reviewer’s comment. We agree that the true replication is the number of fields. In our study, the main purpose is modeling the gene flow. Therefore, the experiment was designed for establishing the regression models.

(9) I can't read the figures, there is too much blemish.

**Response to Reviewers:**

Thanks for the reviewer’s comment. We have modified the resolution of the figures.

Reviewer #2: The authors of this manuscripts present bootstrap simulations for evaluating the model estimation of the extent of cross-pollination in maize at the field-scale level. It is an interesting manuscript that presents an important case study of the co-existence between genetically modified and non-genetically modified systems in an open environment. One struggle i faced while going through the manuscript was the need of more effort in clarity in writing and correcting grammatical mistakes, and succinctly providing enough details of their findings in their manuscript, that is, there were several incidences, where the information were repetitive with no additional information, also excessive use of abbreviation which makes it hard to remember all their full forms and hindering in the flow of the read. Overall, manuscript has an interesting case study and meets this journal standards for publication.

I have listed my specific comments with line numbers below:

L 51: Please provide the citation

**Response to Reviewers:**

Thanks for the reviewer’s comment. The citation was the same as [1].

L 54: 'us' should be 'is'?

**Response to Reviewers:**

Thanks for the reviewer’s comment. The sentence has been corrected.

L 56-57: Its a confusing sentence. Should it be interest or concern?

**Response to Reviewers:**

Thanks for the reviewer’s comment. The sentence has been corrected.

L 77: The word 'pollution' is not here in this sentence. May be use contamination instead?

**Response to Reviewers:**

Thanks for the reviewer’s comment. In the gene flow study, the word ‘pollution’ means gene exchange between different varieties through pollen.

L 133: "Polluted" is not the correct use here

**Response to Reviewers:**

Thanks for the reviewer’s comment. The sentence has been corrected as ‘reduce the risk of conventional crops being hybridized by GM crops’ in LINE 133.

L 147-149: Can cross-incompatibility trait assist in resolving this issue? For example check this paper -- Evans, M., Kermicle, J. Teosinte crossing barrier1, a locus governing hybridization of teosinte with maize. Theor Appl Genet 103, 259–265 (2001). <https://doi.org/10.1007/s001220100549>

**Response to Reviewers:**

Thanks for the reviewer’s comment. In our study, the performance of PMFG models is the objective evaluated by the actual field data, not using molecular biotechnology for isolating one category of commercial varieties from another.

L 193-194: the definition about the xenia effect does not make sense. Please correct.

**Response to Reviewers:**

Thanks for the reviewer’s comment. The sentence has been corrected as ‘The xenia effect of the maize is caused by the effect of the different pollen source gene resulted in endosperms on the development of the seeds.’, as shown in LINES 193 to 195.

L 200: Please provide experimental design of the experiments

**Response to Reviewers:**

Thanks for the reviewer’s comment. The detail field design were shown in Fig.1 and noted in LINE 200.

L 217: Out of curiosity, Were there any pollinating insects such as bees that could potentially cross pollinate between two varieties of corn?

**Response to Reviewers:**

Thanks for the reviewer’s comment. Maize is anemophilous flower crop. Therefore, the probability of cross pollinate between two varieties of corn by the pollinating insects is much less than by the wind.

L 220-221: Why only this duration of time was chosen?

**Response to Reviewers:**

Thanks for the reviewer’s comment. In Taiwan and most Asian countries, crop fields are often separated by intervening roadways. Moreover, in Taiwan, there are two suitable seasons for planting maize begin in May and October, respectively. Therefore, the study decided in these two duration of time.

L 233-234: How many corn ears per plant were counted?

**Response to Reviewers:**

Thanks for the reviewer’s comment. In Taiwan, there might be one to three corn ears per plant. However, the first ear has commodity value. Therefore, the first ear was investigated in our study.

L 241-243: Corn plants at the beginning and at the end of each row have a higher probability of containing CP kernels as they are more exposed than the plants in the middle of the row. I hope this scenario was considered while sampling, since it can biased the analysis and the results. similarly, its always advised to harvest top ear of the corn plant for the same reason.

**Response to Reviewers:**

Thanks for the reviewer’s comment. Indeed, Corn plants at the beginning of each row have a higher probability of containing CP kernels than the plants in the middle or the end of the row. Therefore, when designing the sampling plant, the sampling frequency changed with the distance from the pollen source.

L 260: Please correct the spelling of trial

**Response to Reviewers:**

Thanks for the reviewer’s comment. The word has been corrected in LINE 262.

L 317: In statistical analysis, what were the fixed/random effects in the model?

**Response to Reviewers:**

Thanks for the reviewer’s comment. In the PMFG model, the effect of FB and distance were evaluated, as the random effects in the model.

L 318-319: Will the SAS code be published or deposited in any publicly accessible repositories?

**Response to Reviewers:**

Thanks for the reviewer’s comment. In the future, the SAS code in our study will be provided for researchers in need.

6. PLOS authors have the option to publish the peer review history of their article ([what does this mean?](https://journals.plos.org/plosone/s/editorial-and-peer-review-process#loc-peer-review-history)). If published, this will include your full peer review and any attached files.


If you choose “no”, your identity will remain anonymous but your review may still be made public.


**Do you want your identity to be public for this peer review?** For information about this choice, including consent withdrawal, please see our [Privacy Policy](https://www.plos.org/privacy-policy).

Reviewer #1: No

Reviewer #2: No

[NOTE: If reviewer comments were submitted as an attachment file, they will be attached to this email and accessible via the submission site. Please log into your account, locate the manuscript record, and check for the action link "View Attachments". If this link does not appear, there are no attachment files.]

While revising your submission, please upload your figure files to the Preflight Analysis and Conversion Engine (PACE) digital diagnostic tool, <https://pacev2.apexcovantage.com/>. PACE helps ensure that figures meet PLOS requirements. To use PACE, you must first register as a user. Registration is free. Then, login and navigate to the UPLOAD tab, where you will find detailed instructions on how to use the tool. If you encounter any issues or have any questions when using PACE, please email PLOS at [figures@plos.org](mailto:figures@plos.org). Please note that Supporting Information files do not need this step.
