# Supplementary material for: Bootstrap simulations for evaluating the model estimation of the extent of cross-pollination in maize at the field-scale level
Source: PLoS One. 2021 May 19;16(5):e0249700. doi: 10.1371/journal.pone.0249700 (PMC8133429; doi:10.1371/journal.pone.0249700)
Supplement: Attachment — Submitted filename: Response_to_reviewer(20201211).docx [file pone.0249700.s007.docx]

Reviewer #1: L239: CP% is based on counts and as such is bound to display heterogeneity of variance. Residuals need to be carefully checked if simple nonlinear least squares is used and graphical evidence of a good fit based on residual plots should be provided. Alternatively, if residuals indicate problems, the nonlinear models can be fit using a generalized nonlinear regression package allowing a Poisson or Binomial error distribution to be assumed.

Response: Thanks for the reviewer’s comment. We followed your suggestion and checked the residuals, carefully. It was found that the graphical evaluation of goodness of fit indicated that variance was heterogeneous (Fig. 1a-g).

b

a

d

c

f

e

g

Fig. 1. Residual plot of (a) CP_1_, (b) CP_2_; (c) CP_3_; (d) CP_4_; (e) CP_5_; (f) CP_6_; (g) CP_7_ by ordinary least squares method with normal error distribution.

The fitting result of models with Poisson error distribution indicated a partial improvement of the assumption of homoscedastic (Fig 2a-e). Although the characteristic of pollen dispersal data still makes some dispersions, for the further analysis, the fitting of nonlinear models with Poisson error distribution were used to replace the normal error distribution in our previous manuscript.

b

a

c

d

e

Fig. 2. Residual plot of (a) CP_1_, (b) CP_2_; (c) CP_3_; (d) CP_4_; (e) CP_7_ by maximum likelihood method with Poisson error distribution.

L296: I do not think the model requires observed data on P0: P0 is just another parameter that actually needs to be estimated. Thus, I also do not see a need to impute missing data at the first row of the pollen field.

Response: Thanks for the reviewer’s comment. Because the CP rate at the edge of the pollen recipient field vary with different cultivation scenarios, P0 should be calculated separately from different experiments in this study. The model may have a poor performance, if P0 is estimated using the data combining three experiments. Based on the data collected on-site in Taiwan, a pollen dispersion model suitable for use in Taiwan is constructed. In the future, under the condition that P0 can be calculated, CP% under a certain distance can be evaluated.

L337: What are a and b here? Parameters of the nonlinear models, or are you fitting a regression of observed versus fitted data here and a and b are intercept and slope, respectively?

Response: Thanks for the reviewer’s comment. The a and b are the parameters of the nonlinear models.

I had suggested that field is the experimental unit, so the whole study only has three true replications. the authors acknowledge this in their response, but their main text does not comment on this limitation. I think it would be important to do so.

Response: Thanks for the reviewer’s comment. We have described this limitation at the end of Conclusion (lines 602-605).

Reviewer #2: Authors have addressed all my questions very well. However, the figures in the revised version still had a poor resolution such as axis labels and especially equations on figure 6, please fix them.

Response: Thanks for the reviewer’s comment. We have fixed some figures that axis labels and equations are unclear. In addition, figures were assessed by PACE tool to check the technical requirements. The high-resolution figure file can be download by clicking the link at the upper right of figure page.
