# Supplementary material for: Bootstrap simulations for evaluating the model estimation of the extent of cross-pollination in maize at the field-scale level
Source: PLoS One. 2021 May 19;16(5):e0249700. doi: 10.1371/journal.pone.0249700 (PMC8133429; doi:10.1371/journal.pone.0249700)
Supplement: Attachment — Submitted filename: the_response_to_reviewer_20201223.docx [file pone.0249700.s008.docx]

Reviewer #1: The authors insist that imputation of P0 is nececcary prior to fitting some of their models. I can only re-iterate that I think P0 is a parameter that should be estimated simultaneously with the others in order to obtain a valid inference. The authors could compare the estimates of P0 resulting from this with their imputed value of P0. Also note that P0 in the these models has about the same status as b in some of their simpler models.

Response: Thanks for the reviewer’s comment and remind. Each model used in our study was the empirical model cited from the previous studies. And we want to evaluate the optimal mode suitable for use in Taiwan. As remind by reviewer, we also fitted CP4 by taking P0 as a parameter (see Table below). However, the fitting performance of CP4 with estimated P0 was worse than the fitting performance of CP4 with imputed P0. Therefore, according to the fitting performance we tend to keep the original method in our study. Moreover, as pointed by reviewer, the estimation of b in CP3 was very similar the value of P0 without FB in 2009-2, as shown in Tables 2, 3, and 4 in manuscript. On the other hand, we also used CP4 model by taking P0 into consideration. As shown by the simulation results, the SD of deviance were higher in CP3 than in CP4 using P0. This indicated that CP3 had worse fit and greater variability than CP4 did. Moreover, the average AIC values were similar to the results for deviance in Table 5. As descripted in the manuscript, CP7 and CP4 exhibited the lowest average AIC values, and they had smaller SDs compared with the other models. According to the conclusion, based on the data collected on-site in Taiwan, a pollen dispersion model suitable for use in Taiwan is constructed. In the future, under the condition that P0 can be calculated, CP% under a certain distance can be evaluated and provided for decision making in policy.

**Table Comparison of parameters and fitting performance of CP_4_ with imputed P0 and estimated P0.**

| Model | P0 | a | b | deviance | AIC |
| --- | --- | --- | --- | --- | --- |
| CP_4_ |  | 0.2122 | -0.3905 | 41651 | 59951 |
| CP_4__P*^a^* | 0.6423 | 0.0835 | -0.3886 | 50325 | 68626 |

***a*:** P0 was estimated as a model parameter
