# Supplementary material for: Bootstrap simulations for evaluating the model estimation of the extent of cross-pollination in maize at the field-scale level
Source: PLoS One. 2021 May 19;16(5):e0249700. doi: 10.1371/journal.pone.0249700 (PMC8133429; doi:10.1371/journal.pone.0249700)
Supplement: Attachment — Submitted filename: the_response_to_reviewer_20210118.docx [file pone.0249700.s009.docx]

Reviewer #1:

The authors assert that the deviance is better when P0 is imputed than when it is estimated. This suggests that something is wrong. The authors are using maximum likelihood estimation, so the value for P0 they find by that method should maximize the likelihood, and hence minimize the deviance. This can't possibly be beat by imputing P0 in any way. All of this is assuming, of course, that both times they are using the exact same data and the exact same likelihood function, which is necessary if you want to use deviance or AIC to compare models.

Response: Thanks for the reviewer’s comment. The values of P_0_ that used to establish the model were the actual CP rate at the first row of each furrow. Because, there were some missing values of the CP rate at the first row, the missing values were imputed by the k-NN algorithm for fitting the model. In this revision, we also provided the fitting result that P_0_ was defined as a parameter in the supporting information. Then, the process of the comparison of model was used the same likelihood function (Poisson likelihood function) and the same dataset.

The authors state that they fitted the models as generalized linear models (GLM) but the models as stated in equations (2) to (8) are not generalized linear models. Several of them can be turned into a GLM upon taking the logarithm, and I am assuming this is what the authors have done. This would need to be explained succinctly. The parameter P0 of the model CP4 will be an intercept, but on the log-scale. Note that models CP6 and CP7 are not GLMs so it is not clear what was done to fit these. Since the authors are using SAS, they could use the NLMIXED procedure to fit these model (and other models for comparison to make sure they get the likelihood right). At any rate, it would be good to tell readers how exactly the different models were fitted in SAS.

Response: Thanks for the reviewer’s comment. All models have been taken the logarithm (the log link function) to get the linear predictor of models. In the condition that P_0_ is known, the P_0_s of the CP_4_ and CP_7_ were combined with the offset term of the models. The P_0_ was defined as the log-scale intercept in the fitting result of models with parameter P_0_. The SAS procedure used to fit the models was PROC GENMOD. The detail of model fitting has been added in the subsection of Statistical analysis.

One further issue: The authors assert that they assessed "predictive ability" by deviance and AIC. These are not measures of predictive ability. In cross validation, predictive ability can be computed directly by comparing predicted and observed data in the validation set, e.g. via the correlation.

Response: Thanks for the reviewer’s comment. In Fig. 5, the correlation coefficient between predicted and observed data was showed in the original validation data. We have added the correlation coefficient between predicted and observed data in the Table 5 for the simulated validation sets. Also, the description of deviance and AIC was corrected (lines 335, 346, 450-451, 456-457, 522, 531-532, 545-546).
