# Supplementary material for: Bootstrap simulations for evaluating the model estimation of the extent of cross-pollination in maize at the field-scale level
Source: PLoS One. 2021 May 19;16(5):e0249700. doi: 10.1371/journal.pone.0249700 (PMC8133429; doi:10.1371/journal.pone.0249700)
Supplement: Attachment — Submitted filename: Response to reviewer(20210219).docx [file pone.0249700.s010.docx]

Reviewer #1:

The authors maintain that P0 should be imputed and then treated as an offset in the ML estimation of the GLMs. I can only re-iterate that it is better to estimate P0 when fitting the GLM. The deviance and AIC will be better. Conversely, if they use P0 as an offset, they need to make sure the intercept is taken out of the model, using the NOINT option. As this is not mentioned in the paper, I am not sure the models were fitted correctly, even if one accepts that P0 should be an offset.

Response: According to the Table S1 and Table 2, the models with P0 as an estimated parameter perform worse than models with imputed P0. The P0 was combined with the total grain number and was treated as an offset. The option NOINT was used.

I do not think the authors have properly fitted model CP7 because there is no way to turn this into a GLM, yet the authors used the GENMOD procedure to fit it, and this is a procedure to fit GLMs. The authors asser that " In addition, model CP7 was taken the logarithm after subtracting 0.003." Have they substracted 0.003 from the observed response and then fitted a GLM? In that case, they have not fitted the model properly by ML. And the "data", i.e. the response, is not the same, so they cannot compare the deviances and AIC values with the other models. Again, I can just re-iterate my suggestion to fit the model properly using NLMIXED.

Response: After subtracting 0.003 from the observed response, the CP7 can be transformed into GLM. Because the models don’t include the random effect, using NLMMIXED is not necessary.

If the authors have not actually fitted models CP5 and CP6, but used fits from literature, how did they obtain deciance and AIC?

Response: With a proper likelihood function, the deviance and AIC can be calculated from any model with observations. The CP5 and CP6 was treated as a fitted model in our study.
