# Supplementary material for: Bootstrap simulations for evaluating the model estimation of the extent of cross-pollination in maize at the field-scale level
Source: PLoS One. 2021 May 19;16(5):e0249700. doi: 10.1371/journal.pone.0249700 (PMC8133429; doi:10.1371/journal.pone.0249700)
Supplement: Attachment — Submitted filename: Response_to_reviewer(20210317).docx [file pone.0249700.s011.docx]

Reviewer #1:I think the authors need to reconsider the ML theory underling GLMs. The way they responded to my comments indicates that they have not understood my points. I am a bit at a loss what else I can do to make them understand that their analysis is flawed regarding model CP7, and also regarding the estimation of P0 in some of the other models. I'll give it one last try. I'm really trying to help the authors and make sure they do not publish a flawed analysis.

(1) If you are using ML estimation properly, the deviance can only improve when including P0 in the estimation of the model, as compared to imputing P0 in any way. If you find the opposite, which seems to be the case, this is a sure sign that something is wrong in your analysis.

Subsequently, I am particularly refering to these two statements in the authors' response:

"Response: After subtracting 0.003 from the observed response, the CP7 can be
transformed into GLM. Because the models don’t include the random effect, using
NLMMIXED is not necessary."

"Response: With a proper likelihood function, the deviance and AIC can be calculated
from any model with observations. The CP5 and CP6 was treated as a fitted model in
our study."

(2) Just because you can compute a likelihood doesn't mean you can use it to compare model fits. You can only use the likelihood to compare model fits if the data used to fit the different models is exactly the same. So you can't substract 0.0003 from the response for one model but use the raw data "as is" for the others and then use the AIC or deviance to compare model fits. This is a fundamental fact in likelihood theory, and I would strongly urge the authors to consult their favourite mathematical statistics textbook on this point.

(3) If you substract an arbitrary value from a count, it's no longer a count and so it can't reasonably be assumed to follow a Poisson distribution after this operation. I can only re-iterate that the best to fit model CP7 is to use NMLIXED, leaving all model terms on the right-hand side.

Response: Thanks for the reviewer’s comment. In this edition, the models of CP_5_ and CP_6_ have been removed. The original model CP_7_ was renamed CP_5_ in this edition. All models were re-fitted by PROC NLMIXED with Poisson distribution and re-output the relative Tables and Figs in this revision.
